# Supplementary material for: Self- and Cross-Fusing of Furan-Based Polyurea Gels Dynamically Cross-Linked with Maleimides
Source: Polymers (Basel). 2023 Jan 9;15(2):341. doi: 10.3390/polym15020341 (PMC9861426; doi:10.3390/polym15020341)
Supplement: Supplementary file 1 [file polymers-15-00341-s001.zip › polymers-2103523-supplementary.pdf]

Supplementally Material

Self- and Cross-fusing of Furan-based Polyurea Gels Dynamically Cross-linked with Maleimides

Takuya Kumakura, Kenji Takada and Tatsuo Kaneko*

Graduate School of Advanced Science and Technology, Sustainable Innovation Research Area, Japan Advanced Institute of Science and Technology, 1-1 Asahidai, Nomi, Ishikawa, 923-1292, Japan.
***Correspondence author
Tel.: +81-761-51-1631, FAX: +81-761-51-1635, kaneko@jaist.ac.jp

Scheme S1. Syntheses of polyureas from 2,5-bis(aminomethyl)furan and various diisocyanates.

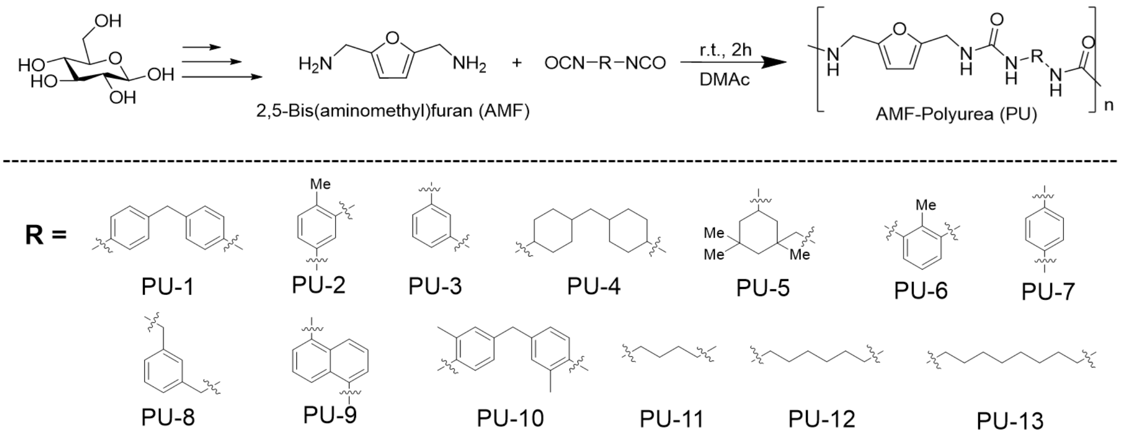

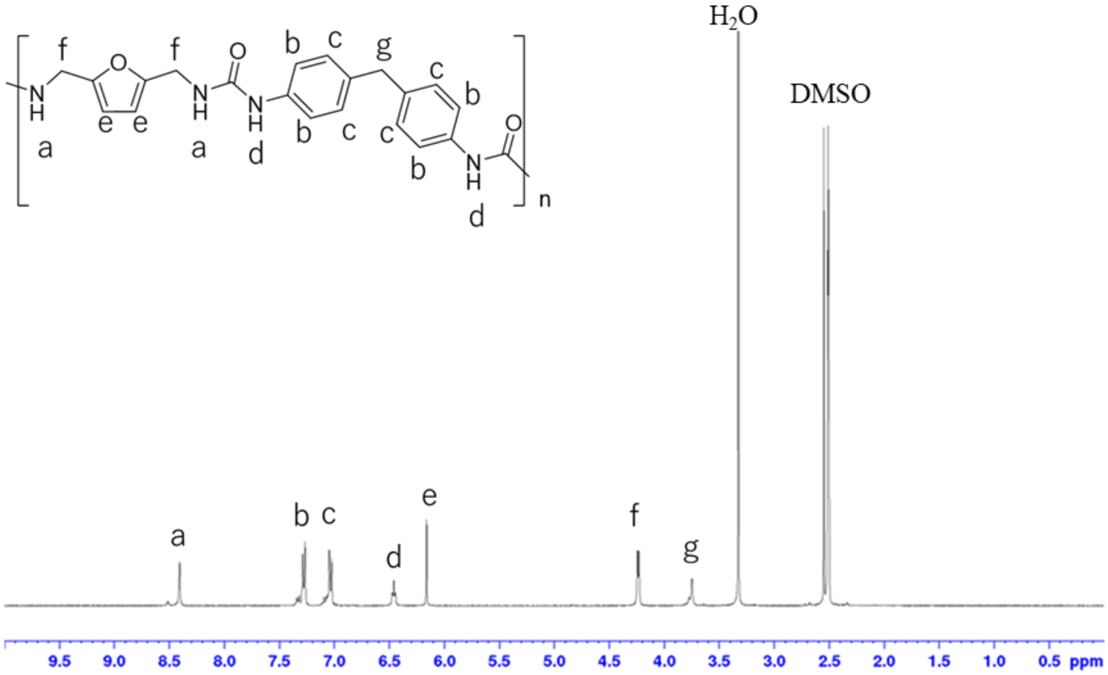

Figure S1 ^{1}H NMR (400 MHz, DMSO-d_6) spectrum of PU-1.

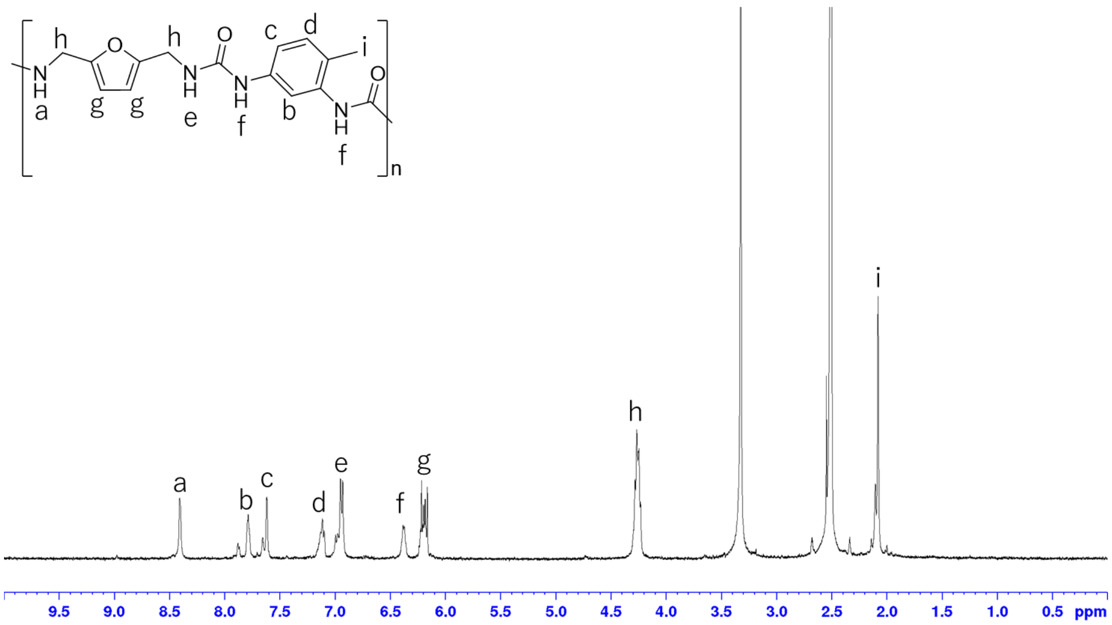

Figure S2 ^{1}H NMR (400 MHz, DMSO-d_6) spectrum of PU-2.

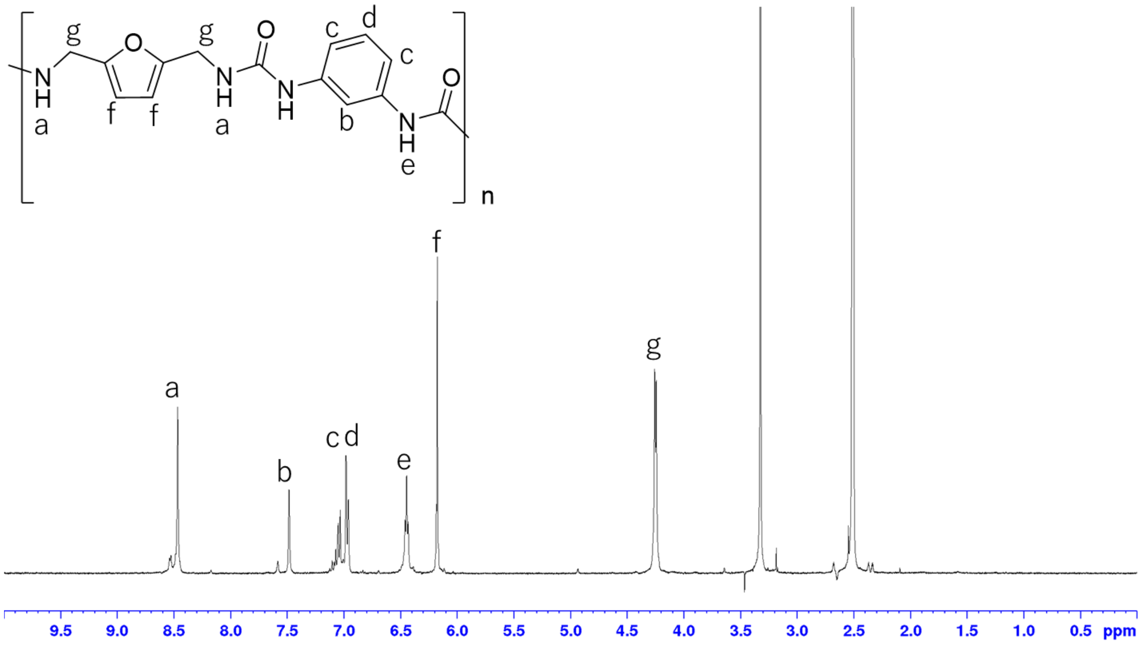

Figure S3 ^{1}H NMR (400 MHz, DMSO-d_6) spectrum of PU-3.

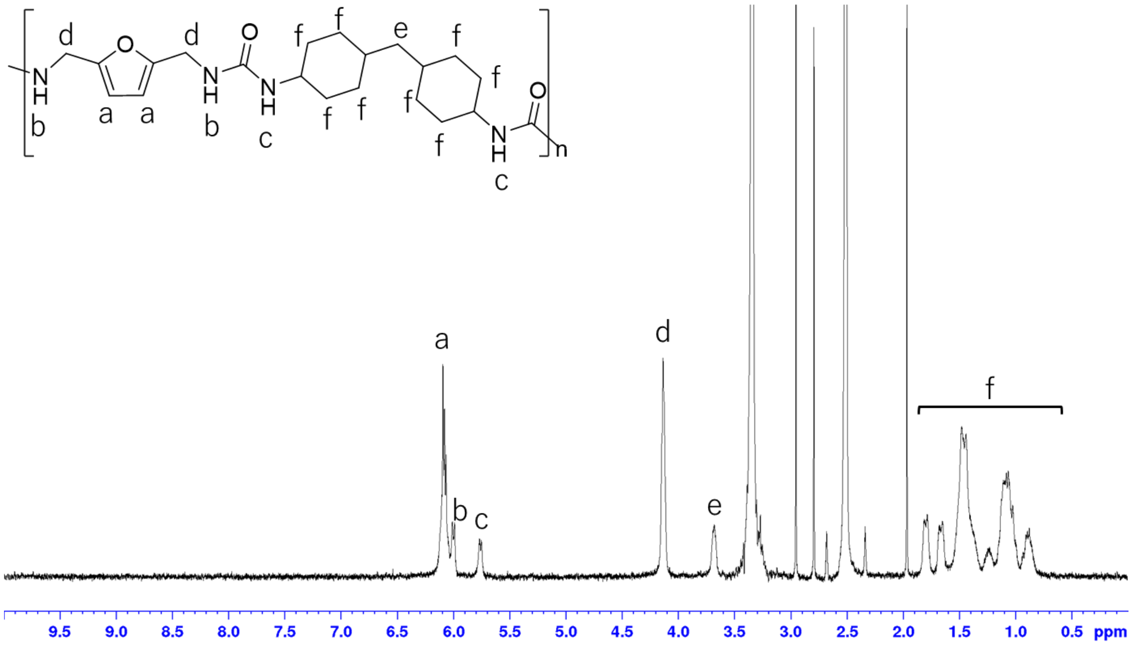

Figure S4 ^{1}H NMR (400 MHz, DMSO-d_6) spectrum of PU-4.

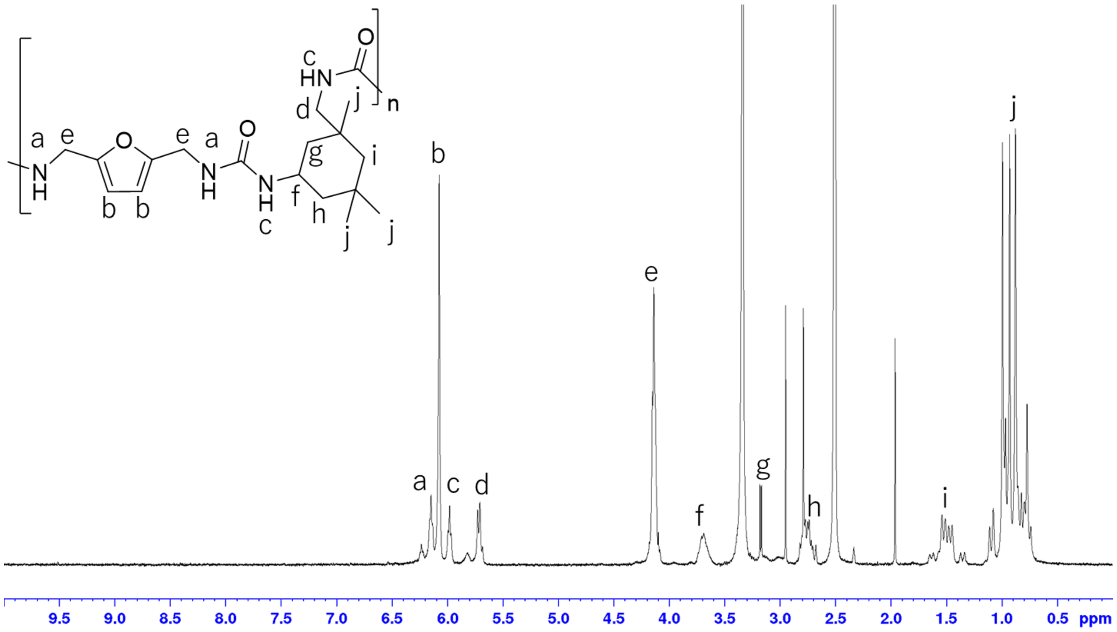

Figure S5 ^{1}H NMR (400 MHz, DMSO-d_6) spectrum of PU-5.

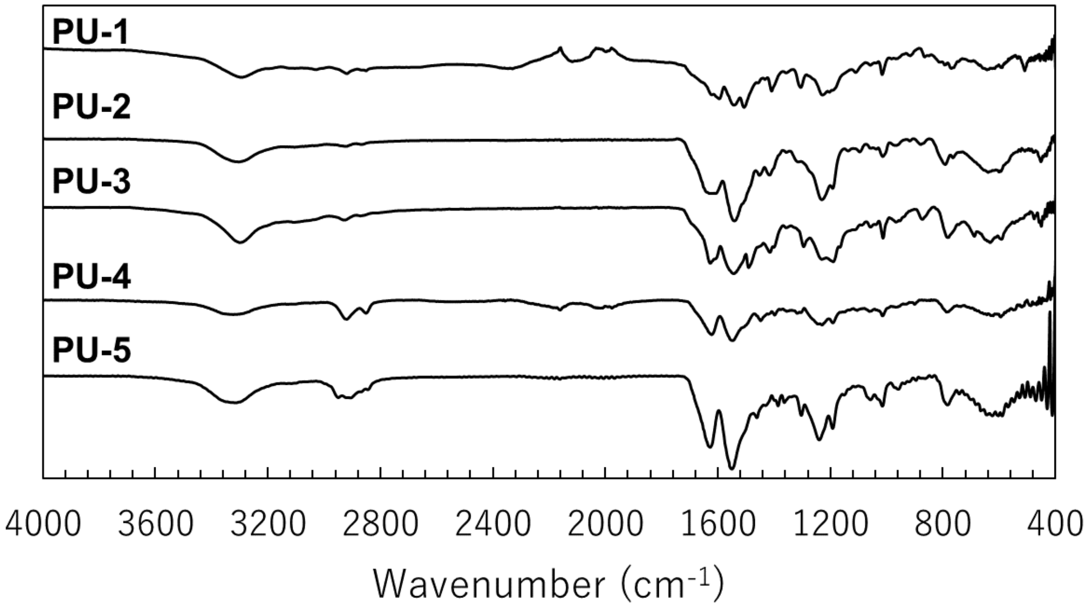

Figure S6 IR spectra of PU-1 to PU-5 using AMF and various diisocyanates.

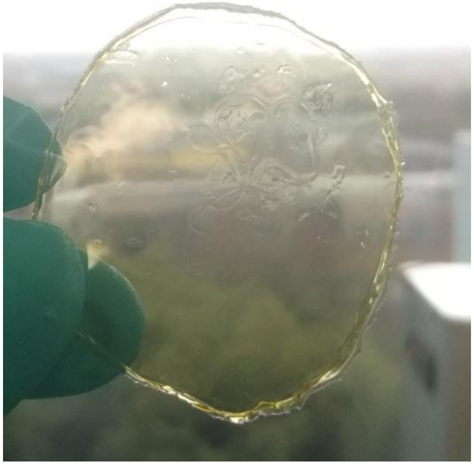

Figure S7 Films of synthesized PU-1 using AMF.

Table S1 Molecular weight, thermal, mechanical, and optical properties of the obtained PU-1 to 5

Polymer	M_n [a]	M_w [a]	M_w/M_n [a]	T_{d5} / °C [b]	T_{d10} / °C [b]	T_g / °C [c]	Strength, σ / MPa	Young's modulus, E / GPa	Elongation at break, ε / %
PU-1	7.4×10^3	2.1×10^4	2.84	280	290	190	65	0.8	8.6
PU-2	16×10^3	94×10^4	6.01	280	290	*N.D.* [f]	25	0.5	6.8
PU-3	8.8×10^3	2.0×10^4	2.39	270	285	175	38	0.7	8.4
PU-4	8.0×10^3	2.0×10^4	2.65	310	320	210	16	0.3	8.9
PU-5	3.0×10^3	1.0×10^4	3.46	300	310	205	5	0.2	2.7

a) Determined by SEC measurement using DMF LiBr solution b) 5 % and 10 % weight loss temperatures, T_{d5} and T_{d10}, were obtained from TGA curve scanned at a heating rate of 10 °C / min under N_2 atmosphere. c) T_g was obtained from DSC curve scanned at a heating rate of 10 °C / min under N_2 atmosphere. d) Not detacted

Table S2 Weight loss temperature and glass-transition temperature of the synthesized PUs using AMF and various diisocyanates.

	T_{d1} (°C) [a]	T_{d5} (°C) [a]	T_{d10} (°C) [a]	T_g (°C) [b]
PU-1	250	280	290	190
PU-2	240	280	290	*N.D.* [c]
PU-3	245	270	285	175
PU-4	290	310	320	210
PU-5	280	300	310	205
PU-6	245	270	280	*N.D.* [c]
PU-7	250	280	290	*N.D.* [c]
PU-8	270	295	300	150
PU-9	250	280	295	*N.D.* [c]
PU-10	255	290	300	*N.D.* [c]
PU-11	270	290	305	115
PU-12	280	300	310	*N.D.* [c]
PU-13	275	300	310	*N.D.* [c]

(a) 5 % and 10 % weight loss temperatures, T_{d1}, T_{d5} and T_{d10}, were obtained from TGA curve scanned at a heating rate of 10 °C / min under N_2 atmosphere. (b) T_g was obtained from DSC curve scanned at a heating rate of 10 °C / min under N_2 atmosphere. (c) Not detected

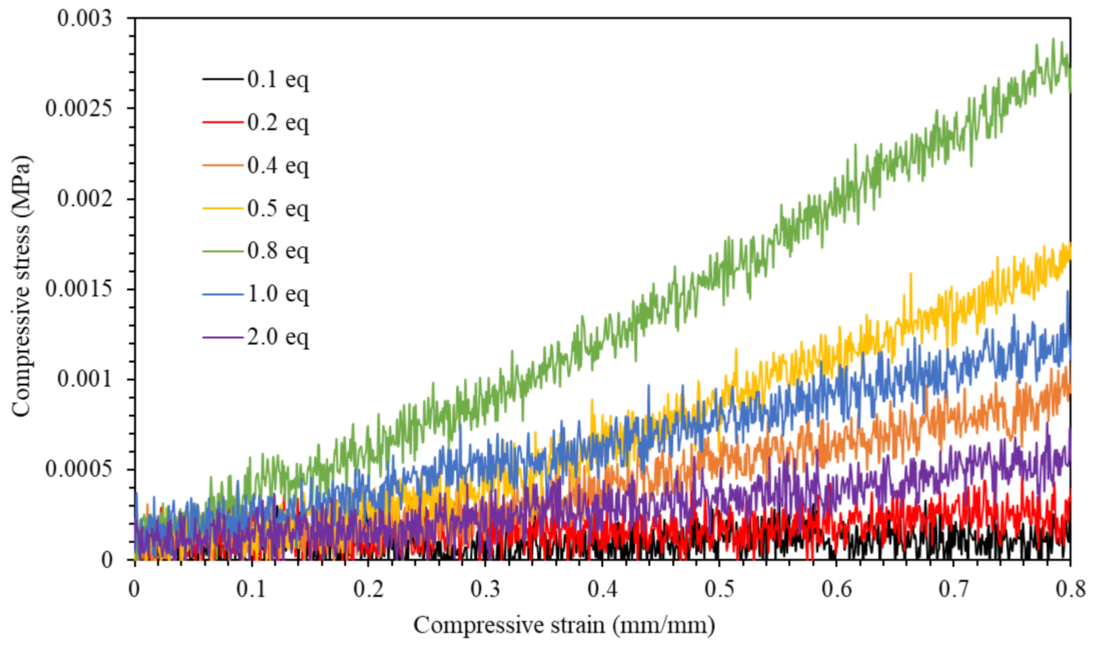

Figure S8 Stress-strain curves of PU-1 gels with different of amount of BMI addition.

Table S3 Mechanical properties of obtained PU-1 gels

Amount of BMI(mol%)	Elastic moduli E_0(kPa)
0.1	0.12 ± 0.01
0.2	0.18 ± 0.05
0.4	0.73 ± 0.04
0.5	0.72 ± 0.07
0.8	0.78 ± 0.2
1.0	0.51 ± 0.03
2.0	0.18 ± 0.04

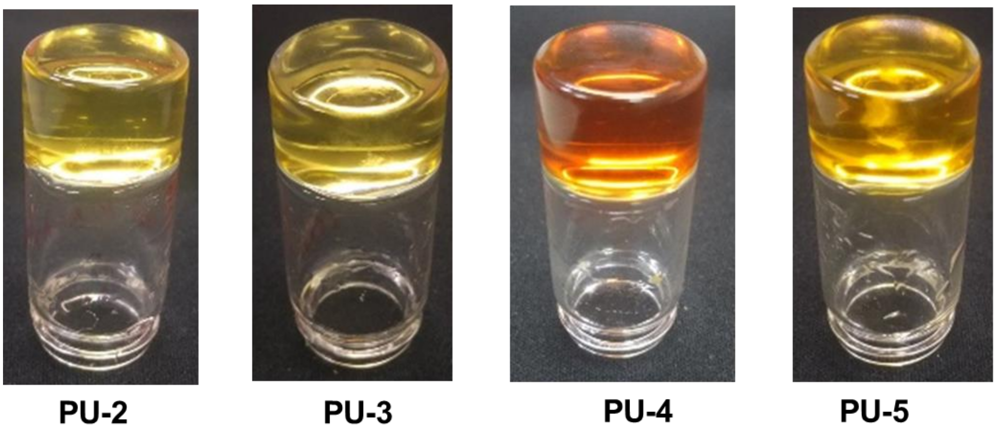

Figure S9 Photographs of gelation of various PUs

Table S4 Mechanical properties of obtained polyurea gels and recovery of mechanical properties by DA reaction.

Amount of BMI(mol%)	Compressive strength, σ(kPa)	Compressive strength after self-healing, σ (kPa)	Compression strain, ε (mm/mm)	Compression strain after self-healing, ε (mm/mm)
10	17.2	23.2	0.9	0.8
50	39.4	60.5	0.6	0.6
80	23.9	15.5	0.5	0.4
100	33.6	38.4	0.6	0.6
